# Genetic Diversity Analysis and Core Collection Development of Indian Mungbean (*Vigna radiata*) Germplasm

**DOI:** 10.3390/plants15111733

**Published:** 2026-06-03

**Authors:** Manickam Dhasarathan, Adhimoolam Karthikeyan, Santhi Madhavan Samyuktha, Lekshmi Jeeva Kasi Vishwanathan, Gunasekaran Ariharasutharsan, Natesan Senthil, Muthaiyan Pandiyan

**Affiliations:** 1 DST-Centre of Excellence for Climate and Disaster Resilient Agriculture, Agro-Climate Research Centre, Tamil Nadu Agricultural University, Coimbatore 641003, India; plantdr.dhasarathan@gmail.com; 2 Centre of Innovation, Agricultural College and Research Institute, Tamil Nadu Agricultural University, Madurai 625104, India; 3 Subtropical Horticulture Research Institute, Jeju National University, Jeju 690756, Republic of Korea; 4 Department of Plant Breeding and Genetics, Agricultural College and Research Institute, Tamil Nadu Agricultural University, Madurai 625104, India; 5 Department of Entomology, Centre for Plant Protection Studies, Tamil Nadu Agricultural University, Coimbatore 641003, India; 6 Department of Plant Molecular Biology and Bioinformatics, Centre for Plant Molecular Biology and Biotechnology, Tamil Nadu Agricultural University, Coimbatore 641003, India; 7 Dr. M.S. Swaminathan Agricultural College and Research Institute, Tamil Nadu Agricultural University, Echangkottai, Thanjavur 614902, India

**Keywords:** agronomic traits, core collection, greengram, pulses, *Vigna* spp.

## Abstract

Mungbean is an important legume crop native to India. In this study, 500 indigenous mungbean accessions collected from diverse eco-geographical regions of India were evaluated for agronomic trait genetic variability and core collection development. The accessions were grown in an augmented design during 2019 and 2020, and data were recorded for seven quantitative and 13 qualitative traits. Analysis of variance (ANOVA), frequency distribution, and box-plot analyses revealed substantial phenotypic variation among the accessions. Traits including plant height (PHT), number of pods per plant (NPP), hundred-seed weight (HSW), and single-plant yield (SPY) exhibited high heritability coupled with high genetic advance, indicating the predominance of additive genetic effects. Principal component analysis showed that the first three principal components explained 70% of the total phenotypic variation. The Shannon–Weaver diversity index further indicated high levels of genetic diversity within the population. Based on quantitative traits, the accessions were grouped into six major clusters and 42 sub-clusters, with SPY, NPP, HSW, PHT, and days to 50% flowering (DFF) contributing substantially to genetic divergence. Correlation analysis suggested that direct selection for SPY and indirect selection through associated traits, including NPP, HSW, PHT, NSP, and pod length (POL), may enhance yield improvement. The germplasm collection also possessed desirable traits such as high yield potential, contrasting maturity groups, and plant types suitable for mechanical harvesting and bold-seeded type. A representative core set comprising 50 accessions was developed using the PowerCore program, providing valuable genetic resources for mungbean breeding and genetic improvement programs.

## 1. Introduction

Mungbean, also known as greengram (*Vigna radiata*), is an important and inexpensive source of dietary protein and a traditional food crop. It is rich in vitamin C, water-soluble antioxidants, and minerals compared to other legumes. Mungbean is cultivated in tropical and subtropical regions as a main crop, backyard crop, bund crop and intercrop across Asia and other mungbean-growing countries. Mungbean plays a crucial role in maintaining soil health because it fixes nitrogen through symbiotic associations, even under low-soil-fertility conditions, and helps reduce soil erosion [1,2]. Therefore, it is considered vital for subsistence agriculture. Mungbean is an autogamous diploid crop with 11 chromosome pairs and a genome size of 543 Mb [3]. Globally, mungbean is cultivated on approximately 7.3 million hectares, with an annual production of around 5.3 million tons [4]. India, considered the primary center of diversity for mungbean [5], has the largest cultivation area (48.52 lakh ha) and production (30.85 lakh tons) [6]. Other major mungbean-producing countries include Myanmar, China, Indonesia, Thailand, Kenya, and Tanzania.

Although improved mungbean cultivars possess a genetic yield potential of 2–3 tons per hectare [7,8], their actual productivity is constrained by environmental factors and biotic stresses, including virus, bacterial, fungal diseases, and insect pests [9]. Consequently, the average productivity remains of approximately 721 kg/ha [4], highlighting the need to enhance the genetic potential of the crop to bridge this yield gap. Ongoing breeding programs aim to overcome these constraints by improving nutritional quality and adapting mungbean to newly expanding cultivation environments. However, achieving these objectives requires breeders to access diverse mungbean germplasm resources as source of valuable traits.

To address the challenges associated with crop improvement, substantial efforts have been made worldwide to broaden the genetic base through the collection and conservation of mungbean germplasm. These initiatives have led to the establishment of extensive germplasm collections in national and international gene banks. India’s National Bureau of Plant Genetic Resources has documented more than 11,000 mungbean accessions, including 7453 indigenous and 3588 exotic accessions. Currently, 4438 accessions are conserved in seed gene bank facilities at −18 °C, comprising collections from various Indian states, as well as exotic germplasm originating from 12 countries worldwide [10].

The N. I. Vavilov Research Institute of Plant Industry (VIR), Russia, maintains 863 mungbean accessions collected between the years 1910 and 1927, which are considered among the oldest existing collections after India. The World Vegetable Center, Taiwan, conserves 6700 accessions, as reported in ref. [11], and the USDA conserve 3928 accessions at the University of Georgia, while Thailand, Japan, Austria, and Australia maintain approximately 2250, 1076, 1006, and 1385 accessions, respectively. Globally, more than 43,000 mungbean germplasm accessions are conserved ex situ [9]. As a safety measure, over 9000 mungbean seed samples are duplicated and stored in the Svalbard Global Seed Vault under the black box system through contributions from thirteen national and international genebanks. Several studies have evaluated genetic diversity in mungbean germplasm collections. Gayacharan et al. [12] analyzed genetic diversity in the mungbean gene pool using 1232 germplasm accessions. Azam et al. [13] assessed the genetic diversity among 166 mungbean accessions using multivariate and multi-trait index approaches to identify superior genotypes. Mwangi et al. [14] investigated phenotypic and genotypic diversity for seven Kenyan mungbean accessions. Takahashi et al. [15] conducted a phylogenetic analysis using 71 accessions representing 28 *Vigna* species and three subgenera (*Ceratotropis*, *Plectrotropis*, and *Vigna*) conserved at the National Institute of Agro biological Sciences, Japan. Chen et al. [16] examined the genetic diversity using 157 mungbean bean accessions, including wild species collected from East Asia.

Tamil Nadu Agricultural University (TNAU), India, conserves mungbean accessions at the Ramiah gene bank and the National Pulses Research Centre. Despite the existence of extensive germplasm collections, their use in crop improvement programs remains limited. In general, the accessibility and effective utilization of germplasm collections decrease as the collection size increases. Moreover, screening large germplasm collections for desirable traits is labor-intensive, time-consuming, and costly. Germplasm collection programs may also lead to the over-representation of certain genetic materials, whereas other types of germplasm remain underrepresented. To overcome these limitations, core collections are developed from broader germplasm repositories. A core collection consists of a carefully selected subset of accessions that capture the maximum genetic diversity of a crop species [17,18] with a minimum of redundancy [11,19,20]. Owing to their smaller size, core collections can be more comprehensively evaluated using limited resources, thereby facilitating their efficient utilization in crop-breeding programs. The PowerCore approach has recently been used to develop core sets from large germplasm collections of mungbean, rice, foxtail millet, and wheat [11,21,22,23,24].

In this context, this study aimed to characterize the genetic diversity of indigenous Indian mungbean germplasm using morpho-agronomic traits and to establish a representative core/mini-core collection to facilitate efficient utilization of genetic resources in mungbean-breeding programs.

## 2. Results

### 2.1. Mean Performance and Analysis of Variance

**Figure 1:**
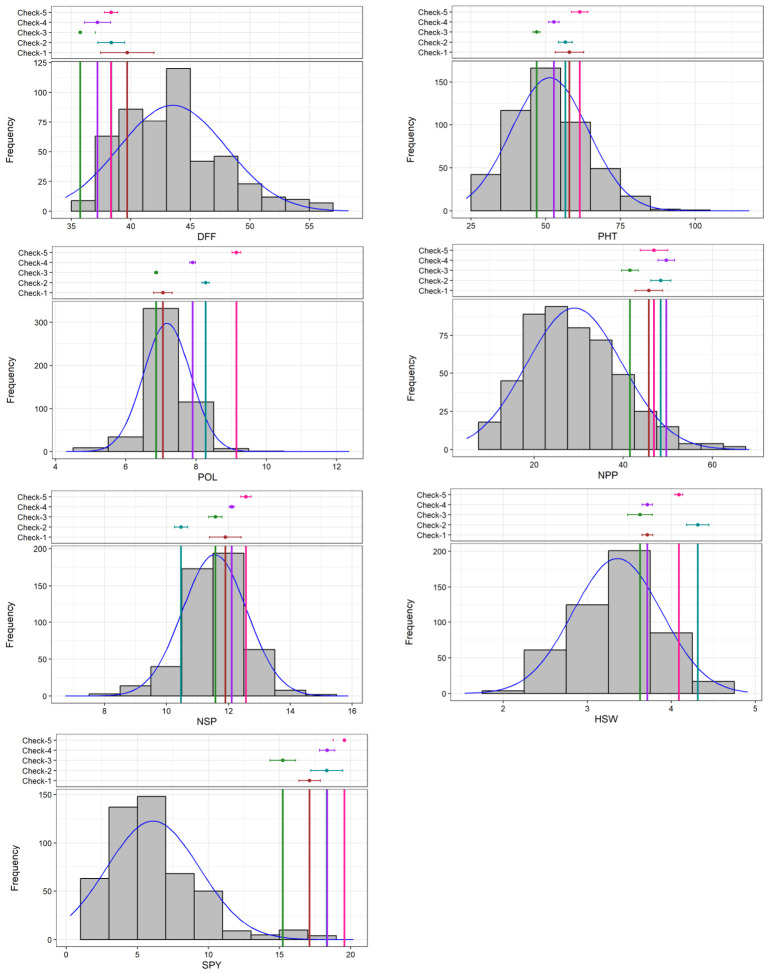
Frequency distribution of quantitative traits in indigenous mungbean germplasm. Note: DFF, days to 50% flowering; PHT, plant height (cm); POL, pod length (cm); NPP, number of pods per plant; NSP, number of seeds per pod; HSW, hundred-seed weight (g); and SPY, single-plant yield (g). Morphometric values of the check varieties CO 6, CO 7, CO 8, VBN 2, and VBN 3 are represented by vertical lines in maroon, dark cyan, green, dark violet, and deep pink, respectively. Blue curve represents the distribution pattern fitted over the histogram.

Field experiments were conducted over two consecutive years. Initially, analysis of variance (ANOVA) was performed separately for each year to assess inter-annual variation. Subsequently, pooled ANOVA was conducted to evaluate genotype performance across years. The analysis revealed significant genotypic variance for all seven agronomic traits (Table S1), indicating substantial phenotypic variability within the indigenous mungbean accessions. Significant genotype × year interactions were observed for days to 50% flowering (DFF), number of pods per plant (NPP), number of seeds per pod (NSP), hundred-seed weight (HSW), and seed yield per plant (SPY), indicating differential genotype performance across years. The mean performance of accessions and check cultivars is presented in Table S2. The trait DFF ranged from 35 to 57 days, with a mean of 44 days after sowing. The PHT varied from 23.50 cm to 118.00 cm, whereas POL ranged from 4.35 cm and 12.38 cm. A wide range of variation was observed for the NPP, which ranged from 6.25 to 66.50 and exhibited a coefficient of variation (CV) of 35.33%. The NSP ranged from 6.75 to 15.75, with a mean value of 11.55. HSW varied between 1.60 g and 4.93 g, with a mean of 3.36 g. SPY ranged from 1.33 g to 19.61 g, with a mean value of 6.10 g, and exhibited high variability (CV = 47.01%) (Table 1). Frequency distribution analysis revealed that HSW exhibited approximately normal distribution (Figure 1).

### 2.2. Genetic Variability and Shannon–Weaver Diversity Indices for Quantitative Traits

Box plots (Figure S1) visually summarized each dataset, illustrating the genetic variability in seven agronomic traits. The phenotypic coefficient of variation (PCV) exceeded the genetic coefficient of variation (GCV) for all traits. PHT, NPP, and SPY exhibited high GCV values (>20%), whereas HSW showed a moderate GCV. Heritability, which indicates the transmissibility of traits from one generation to the next, was high (>60%) for all traits. Genetic advance as a percentage of the mean was significant for PHT, NPP, HSW, and SPY.

The Shannon–Weaver diversity index (H’) was calculated for each trait to evaluate phenotypic diversity in the mungbean indigenous accessions by considering both account allelic richness and the evenness of phenotypic classes. Low H’ values indicate an imbalance among frequency classes and reduced genetic diversity. Diversity values varied across traits, with H’ ranging from 0.698 to 0.859 (mean: 0.808) for all agronomic traits (Table 1), indicating a moderate-to-high level of genetic diversity.

### 2.3. Principal Component and Cluster Analysis for Quantitative Traits

Table 2 presents the percentage of variation explained by each principal component (PC) along with the vector loadings for each trait. The first three PCs accounted for 69.96% of the total variation in the dataset, with each component contributing more than 15% of variability and presenting eigenvalues of 2.32, 1.40, and 1.16, respectively. The remaining PCs explained less than 15% of the variability. PC1, the most influential component, accounted for 33.27% of the total variation. PHT (0.831) and NPP (0.541) contributed the most to PC1, whereas NPP (0.813) and DFF (0.985) contributed the most to PC2 and PC3, respectively. In this study, SPY exhibited the greatest vector length, followed by NPP, HSW, PHT, and DFF, indicating their contribution to total divergence. The angle between trait vectors indicates the nature of association among traits: an acute angle (<90°) suggests a positive correlation, whereas a right angle (90°) indicates no correlation. SPY showed a close association with NPP and HSW. DFF exhibited close vector angles with NSP, PHT, and POL.

**Figure 2:**
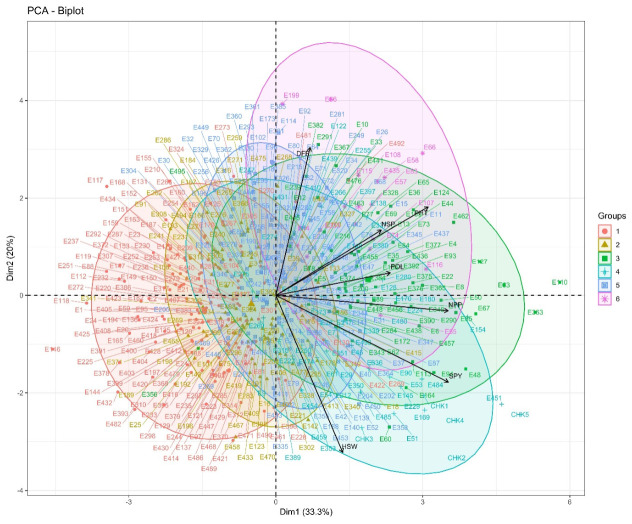
PCA biplot of seven agronomic traits. Note: DFF, days to 50% flowering; PHT, plant height; POL, pod length; NPP, number of pods per plant; NSP, number of seeds per pod; HSW, hundred-seed weight; and SPY, single-plant yield. Dim1 and Dim2 represent the first and second principal components (PC1 and PC2), respectively, obtained from principal component analysis (PCA). Six major groups were identified and are indicated by colored circles

Genotypes in Quadrant 2 exhibited positive loading values for both PC1 and PC2, whereas Quadrant 4 showed positive loading values for PC1 and negative loading values for PC2. Consequently, genotypes positioned in Quadrant II exhibited higher values for DFF, NSP, PHT, and POL. On the other hand, genotypes with superior values for major yield-contributing traits (NPP, SPY, and HSW) were placed in Quadrant 4. Based on the seven agronomic traits, the 500 accessions, including check cultivars, were classified into six major groups (Figure 2).

Group I was the largest cluster, comprising 145 genotypes, followed by Group V with 124 genotypes. Groups II, III, IV, and VI comprised 86, 70, 60, and 15 genotypes, respectively (Table S3). Accessions within each major group were further subjected to hierarchical clustering using the UPGMA method, resulting in the formation of 42 subgroups (Figure S2). Group I was subdivided into seven subgroups, whereas Groups II, III, IV, V, and VI were divided into eight, seven, eight, seven, and five subgroups, respectively. The highest mean values for major agronomic traits were observed in different groups as follows: PHT (86.28 cm), POL (7.63 cm), and NSP (12.23) in Group VI; and NPP (42.88), HSW (3.47 g), and SPY (9.05 g) in Group IV. Early-maturing accessions with a mean DFF value of 42.44 days were predominantly grouped in Group II (Table 3).

### 2.4. Correlation Analysis for Quantitative Traits

**Figure 3:**
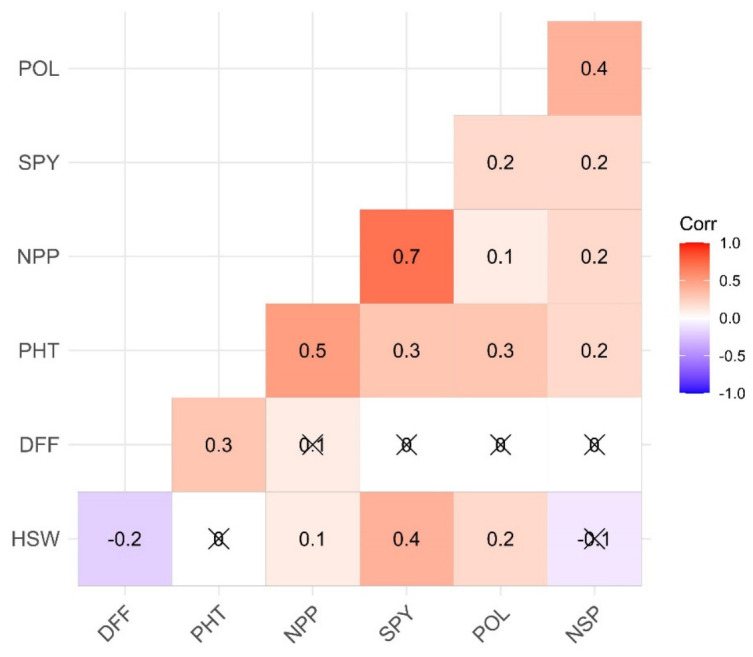
Correlation coefficients among seven agronomic traits in indigenous mungbean collections. **Note:** DFF, days to 50% flowering; PHT, plant height; POL, pod length; NPP, number of pods per plant; NSP, number of seeds per pod; HSW, hundred-seed weight; and SPY, single-plant yield.

Significant and positive correlations were observed among agronomic traits in the indigenous mungbean accessions. Phenotypic correlation analysis of seven agronomic traits identified 15 associations. Correlation coefficients greater than 0.707 or lower than −0.707 were considered biologically significant, as they predict more than 50% of the variation in the corresponding trait [25,26,27]. A strong positive correlation was observed between SPY and NPP (r = 0.7) in the indigenous mungbean accessions. SPY also exhibited positive associations with HSW (r = 0.4), PHT (r = 0.3), NSP (r = 0.2) and POL (r = 0.2). In addition, NPP showed a positive correlation with PHT (r = 0.5), and NSP with POL (r = 0.4). DFF exhibited no significant association with most traits except PHT (r = 0.3). Negative correlations were observed between HSW and both DFF and NSP (Figure 3).

### 2.5. Core Set Development and Validation

**Figure 4:**
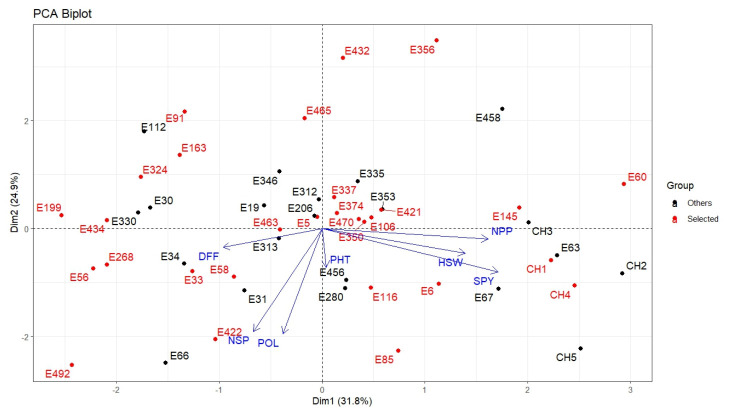
PCA biplot of 50 core indigenous mungbean accessions for quantitative agronomic traits. Accessions shown in red font represent the initial core set comprising 29 accessions generated without manual selection. Accessions shown in black font represent additional entries included in the final core set after applying a 10% selection threshold.

**Figure 5:**
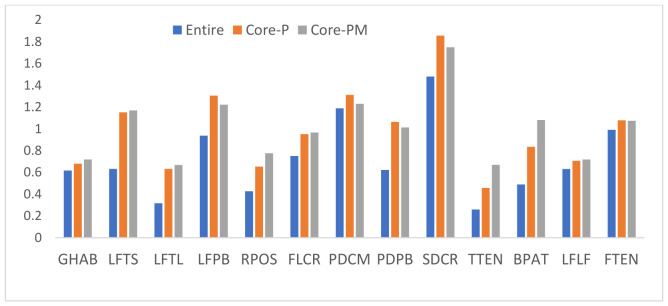
Validation of the core collection in comparison with the entire collection based on the Shannon–Weaver diversity index for qualitative traits. Entire, entire collection; Core_P, core set established using the PowerCore non-heuristic approach; and Core PM, core set established using the modified approach. Note: GHAB, plant growth habit; LFTS, leaflet shape; LFTL, terminal leaf length; LFPB, leaf pubescence; RPOS, raceme position; FLCR, flower color; PDCM, mature pod color; PDPB, pod pubescence; SDCR, seed color; TTEN, twining tendency; BPAT, branching pattern; LFLF, leafiness; and FTEN, flowering tendency.

The PowerCore package program was employed to develop a core germplasm set from the indigenous mungbean accessions. PowerCore applies the advanced maximization (M) strategy coupled with heuristic search algorithms and incorporates Shannon’s diversity indices for core set selection. Without manual classification or forced selection, the program generated an initial core set comprising 29 accessions, representing approximately 6% of the total collection. However, following the recommendation of maintaining a core population size of approximately 10% of the entire collection [17], the selection threshold was adjusted to 10% without applying additional selection criteria (Table S4), resulting in a final core set of 50 accessions. PCA of the 50 core accessions (Figure 4) revealed a dispersed distribution pattern without distinct clustering, similar to that observed in the entire germplasm collection. The distribution pattern of the initial 29 accessions core set is indicated in red font (Figure 4). The developed core set was subsequently compared to the entire germplasm collection (500 accessions) using multivariate statistical analyses. Mean values of quantitative traits between the entire collection and the core subset were compared using a two-sample *t*-test. No significant differences (α = 0.05) were observed for most traits, except PHT, NPP and SPY between the entire germplasm collection and the core subset. Homogeneity of variances between the two sets was assessed using Levene’s test [28] (Table 4). A histogram comparing the CV between the entire and core collections is presented in Figure S3. Furthermore, the Shannon diversity index estimated for 13 qualitative traits revealed higher diversity values in the core subset, comparable to those observed in the entire collection (Figure 5). Four statistical parameters—MD (%), VD (%), CR (%), and VR (%)—were used to evaluate the representativeness of the core set relative to the entire collection. The core subset exhibited an MD% of 7%, a high CR% of 95.69%, a VD% of 47.76, and a VR% of 132.45%.

## 3. Discussion

Assessment of genetic diversity among germplasm accessions facilitates effective utilization of genetic resources and allele mining. Evaluation of genetic variation enables identification of diverse breeding materials possessing desirable agronomic traits, thereby improving the efficiency of crop improvement programs [29,30]. In the present study, a representative core collection was developed using 500 indigenous mungbean accessions collected from diverse geographic regions of India based on seven quantitative and 13 qualitative traits. Significant genotypic variation observed across the indigenous mungbean germplasm indicates the presence of substantial genetic diversity for important agronomic traits. Pooled ANOVA across two seasons demonstrated significant differences among genotypes for all seven agronomic traits, suggesting considerable scope for selection and genetic improvement. Similar multi-location and multi-season evaluations have been effectively used to characterize genetic variability and identify superior breeding materials [31,32]. Earliness is an important breeding objective in mungbean, particularly under intensive cropping systems and climate-vulnerable production environments. In the present study, five accessions exhibited early flowering (<37 days), indicating their potential utility in developing short-duration cultivars. Notably, accession E454 combined early flowering with higher-than-average SPY, suggesting its potential value as a donor parent for simultaneous improvement in earliness and productivity. Such accessions may facilitate the development of transgressive segregants through recombination breeding. In contrast, Chiteri et al. [33] reported approximately symmetrical distribution for DFF in a mungbean panel comprising 480 accessions. Comparable findings have also been reported by Gayacharan et al. [12], Afroz et al. [34], and Nalajala et al. [35].

A total of 49, 32, 43, 23, 50 and 28 accessions exhibited significantly higher mean values for PHT, POL, NPP, NSP, HSW and SPY, respectively, at the 5% significance level. Accessions E60, E113, E154, E451, E484, E169, E51, E229, CHK2 and CHK5 exhibited significantly higher yield in association with increased HSW and NPP. In addition, accessions E451 and E85 showed significantly higher POL, NSP and SPY. Accessions E164, E421, E490, E489 and E358 were identified as bold-seeded types with HSW values greater than 4.80 g. These superior genotypes may be valuable for multi-location yield trials and could be effectively utilized in breeding programs for trait introgression into elite cultivars. Extensive variation was also observed for 100-seed weight among indigenous mungbean accessions, consistent with previous reports by Schafleitner et al. [11], Gayacharan et al. [12] and Yimram et al. [36]. Frequency distribution analysis indicated that HSW exhibited approximately near-normal distribution patterns, suggesting polygenic inheritance of the trait [33]. Box plot analysis further highlighted substantial phenotypic variation among the seven agronomic traits.

Genetic variability indicates the extent of variation among individuals within a population arising from genetic factors. In the present study, high heritability estimates were observed for all agronomic traits. High heritability coupled with genetic advance as a percentage of the mean was recorded for PHT, NPP, SPY, and HSW, indicating the predominance of additive genetic variance. The results suggest that selection for these traits would be effective and may facilitate the identification of elite parental lines [12,37,38]. The observed variability also indicates the potential for selecting genetically diverse parental lines in hybridization programs to generate superior recombinants for yield-associated traits. Similar findings have been reported previously by Gayacharan et al. [12], Afroz et al. [34], and Nalajala et al. [35]. The Shannon–Weaver diversity index (H’) estimated for seven agronomic traits indicated high phenotypic diversity within the indigenous mungbean collection, with a mean H’ value of 0.808. This level of diversity was comparable to that reported for the World Vegetable Center global collection (0.820) and mini-core collection (0.790). The observed diversity may be attributed to geographical adaptation, accumulation of novel genetic variation, and continuous breeding efforts across different agroecological regions. Similar levels of diversity have also been reported in mungbean and other legume crops by Schafleitner et al. [11], Bisht et al. [39], and Shanmugasundaram et al. [40] and in related studies [27,41,42].

PCA is an effective multivariate approach for assessing genetic divergence by reducing while retaining major sources of phenotypic variation. Standardization of variables ensures equal contribution of individual traits to divergence analysis irrespective of scale differences [43]. In the present study, the first three principal components explained 69.96% of the total phenotypic variation, indicating that a substantial proportion of variability within the indigenous mungbean germplasm could be explained by a limited number of components. Similar levels of variation explained through PCA have previously been reported in mungbean germplasm collections [12,29,36]. The evaluated genotypes were distributed across all four PCA quadrants, reflecting broad phenotypic diversity within the collection. SPY contributed the highest proportion of variation, followed by NPP, HSW, PHT, and DFF, indicating the importance of these traits in determining genetic divergence among accessions. Positive loading values for NPP, HSW, and SPY on both PC1 and PC2 suggest strong positive contributions of these traits toward overall variability. In contrast, DFF, POL, PHT, and NSP exhibited positive loading on PC1 and negative loading on PC2, indicating differential trait associations across principal components [44]. Genotypes associated with higher PC1 scores may therefore represent valuable parental resources for breeding programs targeting yield improvement and broadening of the mungbean genetic base. Similar PCA patterns and trait contributions were reported by Gayacharan et al. [12], Basnet et al. [29], and Yimram et al. [36], demonstrating the effectiveness of PCA for characterization of genetic diversity and parental selection in mungbean-breeding programs.

Hierarchical clustering based on Euclidean distances derived from the first two principal components classified the 500 genotypes, including check varieties into six major groups based on the seven agronomic traits. Similar results have previously been reported in mungbean by Udensi and Edu [45], Patel et al. [46], and Manoj et al. [47]. Early-maturing accessions were predominantly grouped in Cluster II, whereas Cluster I consisted of genotypes characterized by early flowering, shorter plant stature and pod length, fewer pods per plant and seeds per pod, and lower yield potential. Genotypes belonging to Clusters I and II may therefore be useful for developing early-maturing cultivars adapted to environments prone to terminal drought and lodging [48,49]. In contrast, genotypes grouped within Clusters III and IV exhibited superior agronomic performance, including higher yield, bold seed type, increased seed number per pod, higher pod number, and tall plant [50]. These accessions may serve as valuable parental resources for generating high-yielding recombinants and transgressive segregants. In particular, genotypes from Cluster IV may be useful for simultaneous improvement in earliness and yield. Cluster VI consisted predominantly of tall and small-seeded genotypes [51,52], which may be useful for developing ideotypes suitable for mechanical harvesting. In addition, smaller seed size has been associated with reduced oviposition preference of bruchid beetles, suggesting the potential utility of these accessions in breeding programs targeting bruchid resistance [53,54]. Cluster III also contained high-yielding erect plant types suitable for mechanical harvesting [55]. Similar grouping patterns have previously been reported by Patel et al. [45] and Mohanlal et al. [56]. Further, hierarchical clustering of accessions within each major group using the UPGMA method resulted in the formation of 42 subgroups. Cluster I was subdivided into seven subgroups, whereas Clusters II, III, IV, V, and VI were further divided into eight, seven, eight, seven, and five subgroups, respectively. The observed subgrouping pattern indicated the presence of substantial diversity both among and within clusters. Accessions grouped within the same cluster or subgroup may possess close genetic relationships, potentially reflecting shared ancestry, ecotypic adaptation, or common geographical origin. Similar observations have been reported previously in mungbean germplasm collections by Gayacharan et al. [12], Sen and De [57], and Goyal et al. [58].

Phenotypic correlation analysis among seven agronomic traits in the indigenous mungbean subset collection revealed several positive associations. SPY exhibited positive correlation with NPP, HSW, PHT, NSP and POL, indicating the importance of these traits in yield determination. In addition, NPP showed a positive association with PHT, whereas NSP was positively correlated with POL. DFF exhibited no significant association with most traits except PHT. Negative correlations were observed between HSW and both DFF and NSP. Similar correlation patterns have previously been reported in mungbean by Azam et al. [12] and Makeen et al. [59]. Development of a representative core collection is essential for improving the efficiency of characterization, evaluation, and utilization while retaining maximum genetic diversity from the entire collection [60]. In this study, a core germplasm collection consisting of 50 accessions was developed using the advanced M (maximum) strategy with a heuristic search algorithms. The developed core set included elite accessions such as E60, CHK2 and CHK5, which represents superior performance for SPY in association with higher HSW and NPP values. Similarly, accessions E85 was included as a representative genotype for POL, NSP, and SPY. Accession E421, identified as a bold-seeded genotype with HSW exceeding 4.80 g, was also retained in the final core collection. These results indicate that the developed core subset effectively captured agronomically important variation present within the indigenous mungbean germplasm.

Validation statistics were computed to assess the representativeness of the developed core germplasm collection. During core set validation, two-sample *t*-tests revealed significant differences (*p* < 0.05) for PHT, NPP, and SPY between the entire and core collections, indicating that the core collection did not fully reproduce the mean values of the original population. Similar observations were reported by Dutta et al. [23], who identified heterogeneity of variance for seven out of 15 traits between the core and entire collections in wheat. Such differences may be attributed to the substantially larger number of accessions represented in the entire collection relative to the core subset. However, the primary objective of core collection development is not to reproduce identical population means, but rather to maximize genetic diversity while minimizing redundancy within a manageable subset. To further evaluate the efficiency and representativeness of the developed core collection, four statistical parameters—MD (%), VD (%), CR (%), and VR (%)—were estimated to compare the mean and variance distributions between the core and entire collections.

Core collections are representative of the original collection when two criteria are satisfied: (1) fewer than 20% of traits exhibit significant differences in mean values between the core and entire collections at *α* = 0.05, and (2) the coincidence rate (CR) remains above 80% across evaluated traits [61]. In the present study, MD was below 20%, whereas the coincidence rate exceeded 80%, indicating that the developed core collection adequately represented the genetic diversity present in the entire collection. In addition, higher VD% and VR% values further suggested that the core subset effectively captured the extent of phenotypic variation within the original germplasm. Similar observations have previously been reported in mungbean and other crop core collections by Schafleitner et al. [11], Ambati et al. [24], Bisht et al. [39], Hu et al. [61], Liu et al. [62], and Moe et al. [63]. In morphological characterization studies, descriptors and descriptor states are generally considered analogous to loci and alleles, respectively. In this study, allelic evenness was assessed using the Shannon–Weaver diversity index (H’), whereas allelic richness was estimated based on the number of descriptor states for each qualitative trait without considering their individual frequencies. Comparison of H’ values between the core and entire collections for 13 qualitative traits revealed higher diversity indices in the core subset, indicating improved allelic evenness and effective representation of phenotypic diversity within the developed core collection.

In summary, the present study revealed substantial genetic diversity among indigenous mungbean accessions for important agronomic traits. Superior genotypes identified for yield, bold seed, early maturity, and plant architecture suitable for mechanical harvesting represent valuable breeding resources. Direct selection for SPY, together with indirect selection through associated traits such as NPP, HSW, PHT, NSP, and POL, may improve breeding efficiency. Cluster analysis identified diverse genetic groups useful for developing broad-based breeding populations. The developed core collection provides an important genetic resource for trait-specific selection, allele mining, and future mungbean genetic improvement programs.

## 4. Materials and Methods

### 4.1. Plant Genetic Materials and Study Site

A total of 500 indigenous mungbean accessions conserved at Ramiah gene bank and National Pulses Research Centre, Tamil Nadu Agricultural University (TNAU), India, were used in this study (Supplementary Table S1). The collection comprised obsolete cultivars, modern cultivars, advanced breeding lines, and mutant lines collected from diverse agro-climatic regions of India, including New Delhi, Punjab, Tamil Nadu, Uttar Pradesh, Maharashtra, Haryana, Andhra Pradesh, Gujarat, and Rajasthan. Field experiments were conducted during the kharif seasons (June–August) of 2019 and 2020 at the Experimental Farm, Agricultural College and Research Institute, TNAU, Eachangkottai (10.66, 79.15).

### 4.2. Experimental Design and Traits Evaluation

The experiment was conducted using an augmented randomized complete block design comprising three blocks to assess genetic variability and divergence among 500 indigenous mungbean accessions along with five standard checks. Each experimental plot consisted of a 4 m row with a spacing of 30 cm between rows and 10 cm between plants. Standard crop management and plant protection practices were followed according to the TNAU crop production guide [64]. For each accession, five competitive plants were randomly selected for recording observations on days to 50% flowering (DFF), plant height (PHT; cm), pod length (POL; cm), number of pods per plant (NPP), number of seeds per pod (NSP), hundred-seed weight (HSW; g) and single plant yield (SPY; g). In addition, 13 qualitative traits were recorded: plant growth habit (GHAB; erect = 1, semi erect = 2, and spreading = 3), leaflet shape (LFTS; entire = 1 and lobed = 2), terminal leaf length (LFTL; small = 3, medium = 5, long = 7), leaf pubescence (LFPB; glabrous = 1, pubescent = 3), raceme position (RPOS; above canopy = 1, intermediate = 2, No visible pod above canopy = 3), flower color (FLCR; yellow = 1, greenish yellow = 2, yellowish green = 3, green purplish yellow = 4), pod color mature (PDCM; straw = 1, tan = 2, brown = 3, brown and black = 4, black = 5), pod pubescence (PDPB; glabrous = 0, puberulent = 3, moderate = 5, dense = 7), seed color (SDCR; light green = 1, dark green = 2, greenish yellow = 3, yellow = 4, brown = 5, dark green mosaic = 6, brownish green = 7, bluish green = 8, brown mosaic = 9), twining tendency (TTEN; none = 0, slight = 3, intermediate = 5, pronounced = 7), branching pattern (BPAT; central 1, basal = 2, top = 3, all over = 4), leafiness (LFLF; sparse = 3, intermediate = 5, abundant = 7), flowering tendency (FTEN; synchronous = 3, intermediate = 5, asynchronous = 7) [65].

### 4.3. Statistical Analysis

Shannon–Weaver diversity indices (H′) [66] for quantitative traits were calculated using mean phenotypic data in Microsoft Excel as follows: H’ = [−∑(n/N) × {log2(n/N)] × (−1)}]/log2k, where H’ represents the standardized Shannon–Weaver diversity index, ‘k’ denotes the number of phenotypic classes for a given trait, ‘n’ represents the frequency of a particular phenotypic classes for a given trait, and ‘N’ is the total number of observations for that trait. For agronomic traits, the accessions were classified into 10 phenotypic classes based on the following class intervals: <*x* − 2sd, *x* − 2sd, *x* − 1.5sd, *x* − sd, *x* − 0.5sd, *x*, *x* + 0.5sd, *x* + sd, *x* + 1.5sd, *x* + 2sd, and >*x* + 2sd, where ‘*x*’ represents the mean and ‘sd’ represents the standard deviation. Analysis of variance (ANOVA) was conducted and the augmented block design was analyzed using the linear model Y_ij = μ+B_i + T_j + ε_ij, where block effects were estimated using replicated checks and used to adjust treatment means. The analysis follows the methodology proposed by Roger G Federer [67] and further described in Statistical Procedures for Agricultural Research [68]. The genetic variability parameters were computed using the methods adopted by Jhonsan et al. [69].

Descriptive statistics, including range, coefficient of variation (CV), standard deviation (SD), mean, standard error of mean (SE), and box plots, were developed by employing the R script “augmented RCBD” [70]. Phenotypic diversity in germplasms accessions were assessed through hierarchical clustering based on Euclidean distance matrices derived from the scores of the first two principal components. Ward’s minimum variance method (ward.D2) was employed for cluster formation, and the dendrogram was partitioned into six distinct groups among the 500 genotypes, including check cultivars, based on seven agronomic traits. The clustering pattern was further visualized using PCA biplots [71,72,73]. The major groups were subsequently subdivided using the Unweighted Pair Group Method with Arithmetic Mean (UPGMA) based on Euclidean distance. Correlation coefficients were calculated using R Script (Rvs4.2) with the “ggplot2” package and SPSS (Version 29.0). Core sets were developed using the PowerCore program (http://genebank.rda.go.kr/powercore.do) (accessed on 30 October 2024), developed by the Rural Development Administration (RDA), South Korea [74]. By default, PowerCore classifies continuous variables into categories according to Sturges’ rule [75], expressed as K = 1 + log2 (n), where n represents the number of observed accessions. The Shannon–Weaver diversity index (H’) was calculated using the phenotypic frequencies to estimate phenotypic diversity for 13 qualitative traits. To evaluate the representativeness of the core collection relative to the entire collection, four statistical parameters were computed: mean difference percentage (MD%), variance difference percentage (VD%), coincidence rate (CR%), and variable range (VR%), as outlined by [22,57,66]. Principal component analysis (PCA) of quantitative traits within the core collections was performed using the “FactoMineR” and “factoextra” in R package.

## Figures and Tables

**Table 1:** Descriptive statistics, genetic variability parameters and Shannon–Weaver diversity indices.

Trait	Min	Max	Mean	SE	CV (%)	Skewness	Kurtosis	PCV	GCV	H^2^_b_	GAM	H’
DFF	35.35	57.00	43.51	0.17	8.68	0.85 *	3.61 *	8.69	7.87	81.93	14.69	0.698
PHT	23.50	118.00	51.35	0.57	25.02	0.74 *	4.39 *	25.00	21.97	77.24	39.83	0.854
POL	4.35	12.38	7.17	0.03	9.31	0.54 *	12.06 *	9.30	8.15	76.92	14.75	0.765
NPP	6.25	66.50	29.03	0.46	35.33	0.66 *	3.63 *	35.11	32.05	83.30	60.34	0.859
NSP	6.75	15.75	11.55	0.05	9.05	−0.21	5.45 *	9.05	7.39	66.81	12.47	0.822
HSW	1.60	4.93	3.36	0.02	15.52	−0.04	3.38	15.50	14.10	82.78	26.46	0.841
SPY	1.33	19.61	6.10	0.14	47.01	1.30 *	5.538	46.10	39.61	73.83	70.22	0.817

Note: SE, standard error; CV, coefficient of variation; PCV, phenotypic coefficient of variation; GCV, genotypic coefficient of variation; H^2^_b_, broad-sense heritability; GAM, genetic advance as percentage of the mean; H’, Shannon–Weaver diversity index. DFF, days to 50% flowering; PHT, plant height; POL, pod length; NPP, number of pods per plant; NSP, number of seeds per pod; HSW, hundred-seed weight; SPY, single-plant yield. * indicates significant at *p* ≤ 0.05.

**Table 2:** The eigenvalue, percentage of variation, cumulative percentage, and eigenvector value for the principal components.

Particulars	PC 1	PC 2	PC 3	PC 4	PC 5	PC 6	PC 7
Eigen value	2.329	1.401	1.167	0.829	0.558	0.467	0.248
Variability (%)	33.273	20.014	16.677	11.847	7.978	6.665	3.547
Cumulative (%)	33.27	53.29	69.96	81.81	89.79	96.45	100.00
DFF	0.071	−0.124	0.985	0.096	0.011	0.010	0.016
PHT	0.831	−0.542	−0.127	−0.006	−0.021	−0.008	0.003
POL	0.012	−0.007	−0.016	0.055	0.319	0.911	−0.256
NPP	0.541	0.813	0.082	−0.198	0.001	0.016	0.011
NSP	0.018	−0.001	−0.014	0.029	0.946	−0.298	0.127
HSW	0.001	0.009	−0.027	0.104	−0.044	0.277	0.954
SPY	0.108	0.175	−0.078	0.968	−0.042	−0.071	−0.091

Note: DFF, days to 50% flowering; PHT, plant height; POL, pod length; NPP, number of pods per plant; NSP, number of seeds per pod; HSW, hundred-seed weight; and SPY, single-plant yield.

**Table 3:** Cluster means of mungbean indigenous collections for various quantitative traits.

Clusters	DFF	PHT	POL	NPP	NSP	HSW	SPY
I	42.92	39.22	6.99	18.61	11.11	3.37	4.54
II	42.44	44.76	7.03	28.46	11.62	3.30	5.32
III	45.34	67.61	7.34	41.60	11.74	3.42	8.86
IV	42.45	51.13	7.25	42.88	11.75	3.47	9.05
V	43.93	56.77	7.31	27.89	11.74	3.31	5.51
VI	47.33	86.28	7.63	28.35	12.23	3.31	5.88

Note: DFF, days to 50% flowering; PHT, plant height; POL, pod length; NPP, number of pods per plant; NSP, number of seeds per pod; HSW, hundred-seed weight; and SPY, single-plant yield.

**Table 4:** Validation statistic for core germplasm developed using PowerCore-modified approach.

Traits	Mean		*p* Value	Sig *	Variance		F Value	Sig *	Minimum		Maximum	
	Entire	Core	*t*-test		Entire	Core	Leven’s test		Entire	Core	Entire	Core
DFF	43.51	43.64	0.89	ns	14.55	25.04	3.22	ns	35.76	35.00	37.00	56.00
PHT	51.35	57.80	0.00	s	163.45	332.44	12.16	s	23.50	28.25	30.50	118.00
POL	7.17	7.45	0.39	ns	0.45	1.46	88.26	s	4.35	4.85	4.85	12.38
NPP	29.03	32.50	0.03	s	106.01	171.15	18.87	s	6.25	6.25	10.50	66.50
NSP	11.55	11.42	0.09	ns	1.09	2.16	17.98	s	6.75	6.75	6.75	15.00
HSW	3.36	3.43	0.08	ns	0.27	0.35	0.74	ns	1.60	1.70	1.70	4.86
SPY	6.10	7.60	0.00	s	9.21	23.37	5.82	s	1.33	1.44	1.55	19.56

Note: Core collection developed using the PowerCore program with a modified approach. ‘s’ indicates significance at the 5% level, whereas ‘ns’ indicates non-significance at the 5% level. DFF, days to 50% flowering; PHT, plant height; POL, pod length; NPP, number of pods per plant; NSP, number of seeds per pod; HSW, hundred-seed weight; and SPY, single-plant yield. * indicates significant at *p* ≤ 0.05.

## Data Availability

Data are available from the authors upon request.

## Supplementary Materials

The following supporting information can be downloaded at: https://www.mdpi.com/article/10.3390/plants15111733/s1, Table S1. ANOVA for seven quantitative traits using augmented design in mungbean germplasm; Table S2. Origin and mean performance of mungbean germplasm for quantitative traits along with checks; Table S3. Distribution of mungbean accessions in various clusters; Table S4. Origin and mean performance of core set mungbean germplasm for quantitative traits; Figure S1. Box plot showing distribution of quantitative traits across 500 mungbean indigenous accessions; Figure S2. (a,b) Subgrouping of major group using the UPGMA based Euclidean distance; Figure S3. Coefficient of variation (%) in entire and core collection for different quantitative traits.
