# Supplementary material for: Genetic Diversity Analysis and Core Collection Development of Indian Mungbean (Vigna radiata) Germplasm
Source: Plants (Basel). 2026 Jun 3;15(11):1733. doi: 10.3390/plants15111733 (PMC13258871; doi:10.3390/plants15111733)
Supplement: Supplementary file 1 [file plants-15-01733-s001.zip › 4260851_Supplementary Figures_R1.pdf]

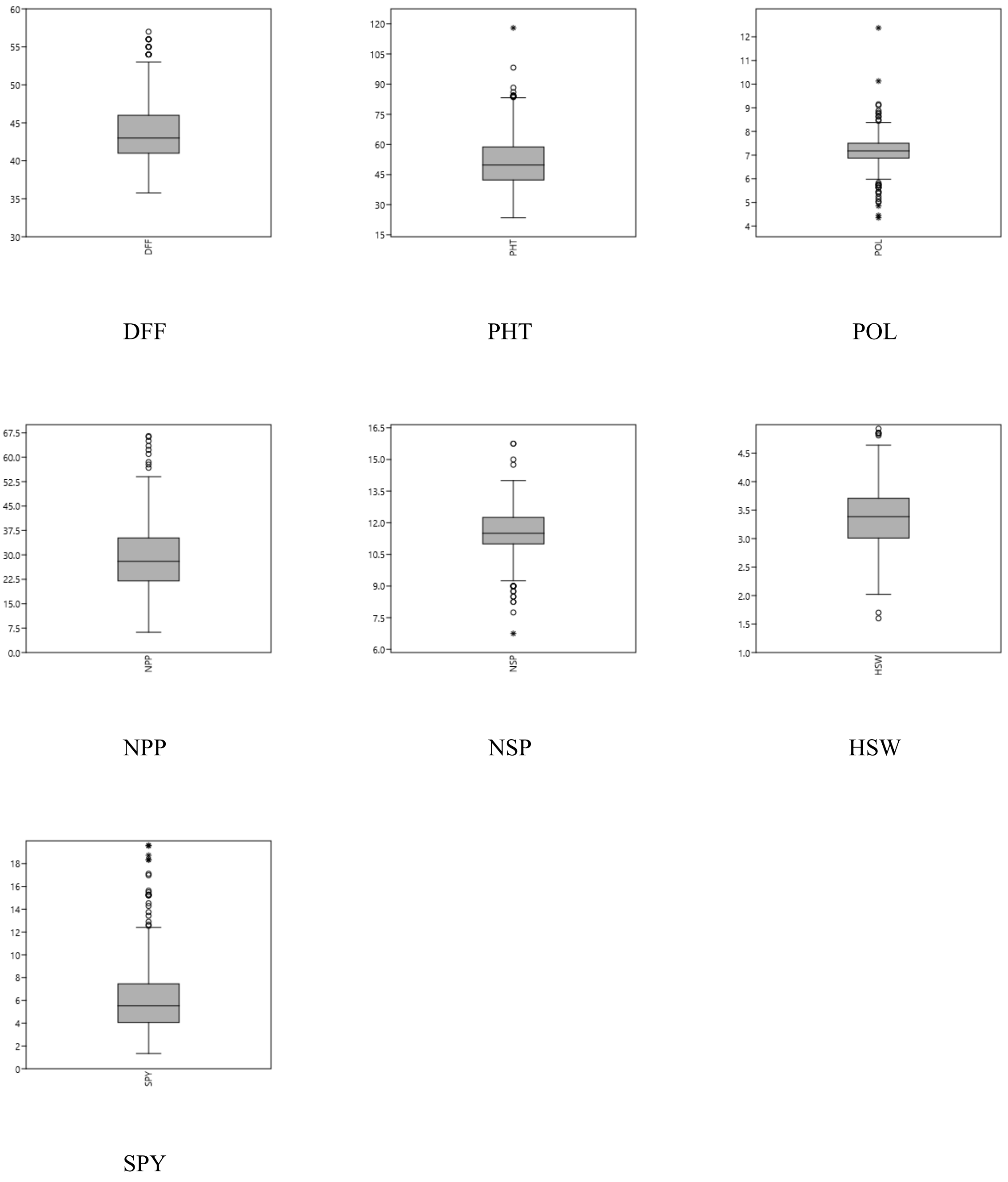

Supplementary Figure S1. Boxplot displaying distribution of 07 quantitative traits across 500 mungbean indigenous accessions. **Note:** DFF, days to 50% flowering; PHT, plant height; POL, pod length; NPP, number of pods per plant; NSP, number of seeds per pod; HSW, hundred-seed weight; and SPY, single-plant yield.

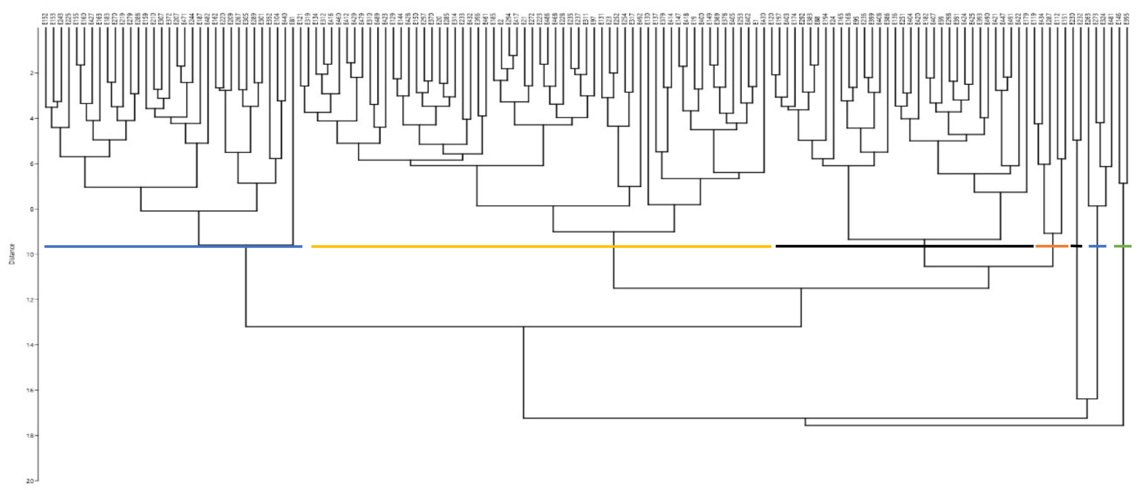

Group 1 further formed 7 sub-group

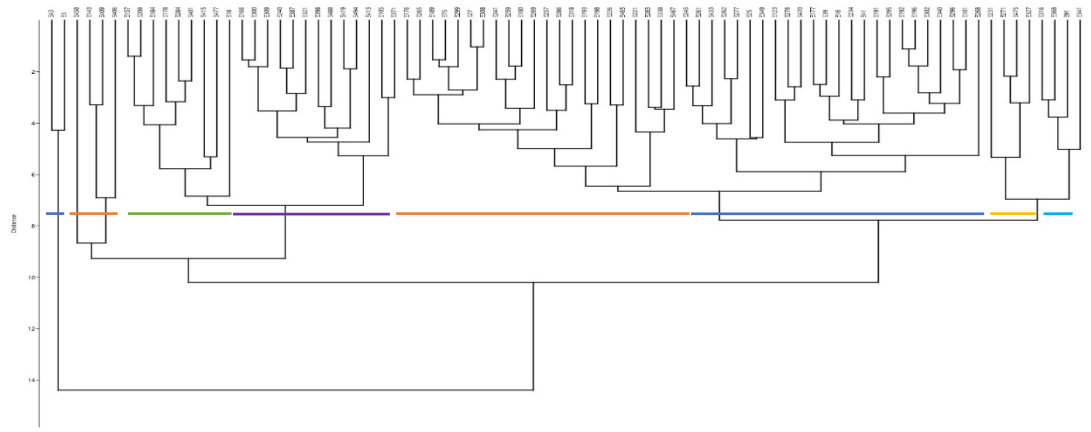

Group 2 further formed 8 sub-group

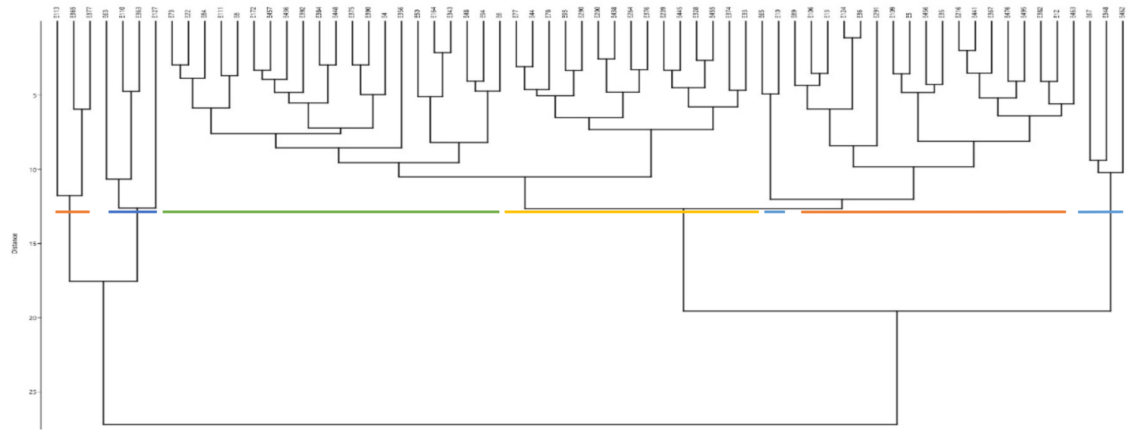

Group 3 further formed 7 sub-group

Supplementary Figure S2a. Subgrouping of major group using the UPGMA based Euclidean distance

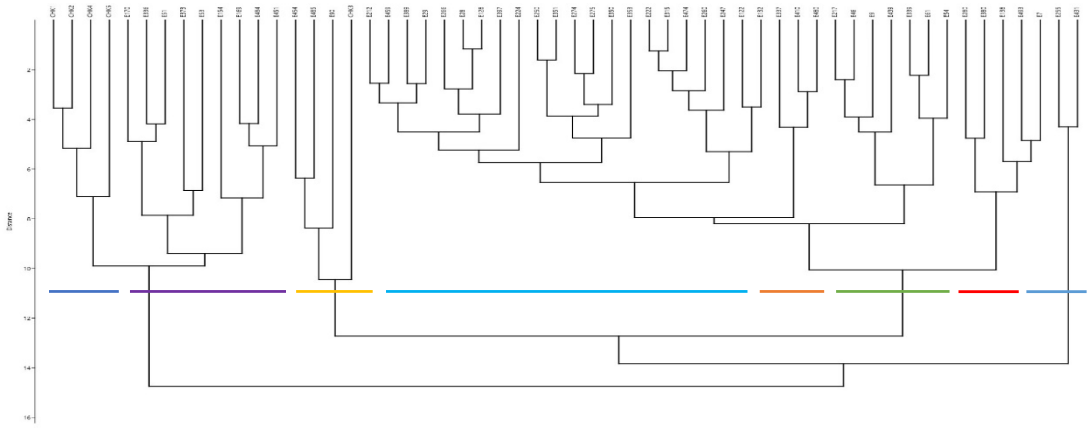

Group 4 further formed 8 sub-group

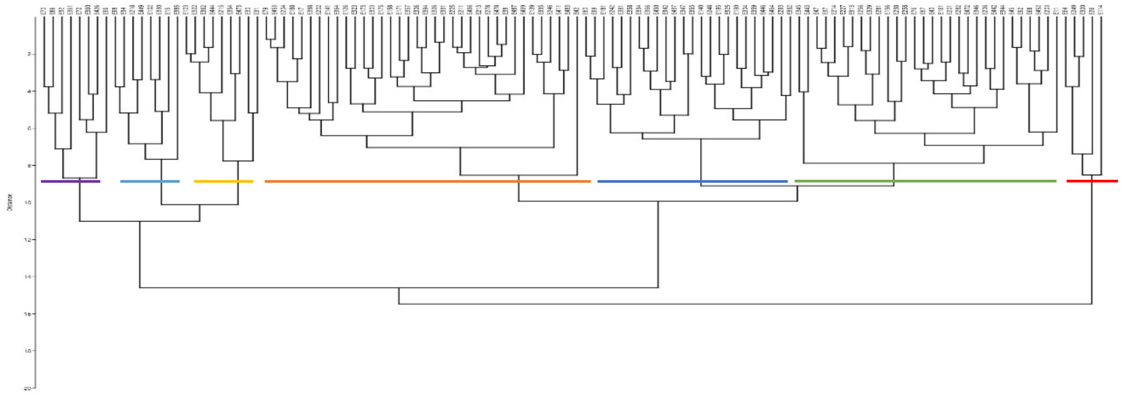

Group 5 further formed 7 sub-group

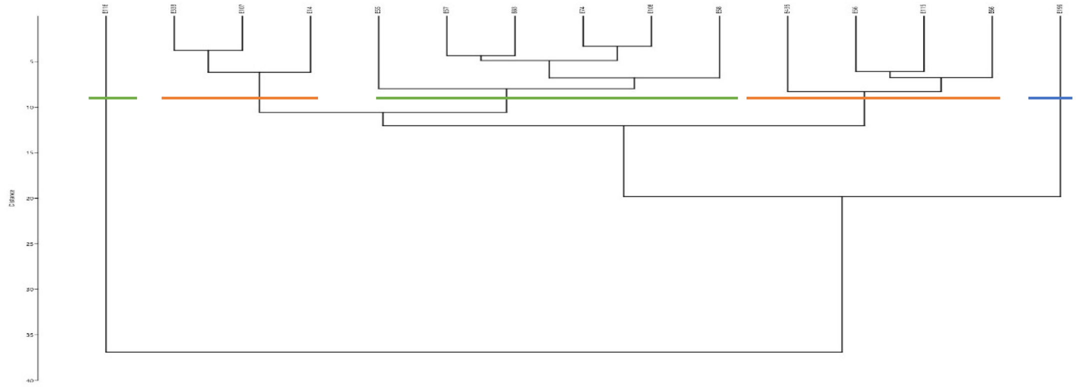

Group 6 further formed 5 sub-group

Supplementary Figure S2b. Subgrouping of major group using the UPGMA based Euclidean distance

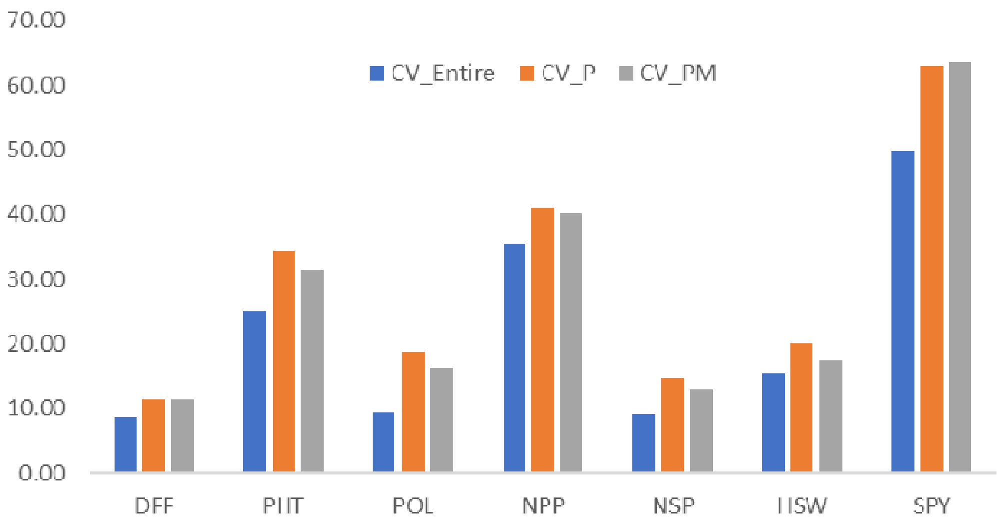

Supplementary Figure S3. Coefficient of variation (%) in entire and core collection for different quantitative traits. **Note:** CV_Entire = coefficient of variation in the entire collection; CV_P = coefficient of variation in the core set established using the PowerCore non-heuristic approach; and CV_PM = coefficient of variation in the core set established using the modified approach. DFF = days to 50% flowering; PHT = plant height; POL = pod length; NPP = number of pods per plant; NSP = number of seeds per pod; HSW = hundred-seed weight; and SPY = single-plant yield.
